# Nationwide trends of hospital admissions for acute cholecystitis in the United States

**DOI:** 10.1093/gastro/gow015

**Published:** 2016-05-11

**Authors:** Vaibhav Wadhwa, Yash Jobanputra, Sushil K Garg, Soumil Patwardhan, Dhruv Mehta, Madhusudhan R. Sanaka

**Affiliations:** 1 Department of Internal Medicine, Fairview Hospital, Cleveland Clinic, OH, USA,; 2 Department of Gastroenterology and Hepatology, Digestive Disease Institute, Cleveland Clinic, Cleveland, OH, USA,; 3 Department of Internal Medicine, University of Minnesota, Minneapolis, MN, USA; 4 Department of Internal Medicine, University of Massachusetts, Worcester, MA, USA; 5 Department of Internal Medicine, Westchester Medical Center, Valhalla, NY, USA

**Keywords:** inpatient admission rates, acute cholecystitis, epidemiology, trends

## Abstract

**Background and aims**: Acute cholecystitis is a fairly common inpatient diagnosis among the gastrointestinal disorders. The aim of this study was to use a national database of US hospitals to evaluate the incidence and costs of hospital admissions associated with acute cholecystitis.

**Method**: We analyzed the National Inpatient Sample Database (NIS) for all patients in which acute cholecystitis (ICD-9 codes: 574.00, 574.01, 574.30, 574.31, 574.60, 574.61 or 575.0) was the principal discharge diagnosis from 1997 to 2012. The NIS is the largest all-payer inpatient database in the United States and contains data from approximately 8 million hospital stays each year. The statistical significance of the difference in the number of hospital discharges, lengths of stay and associated hospital costs over the study period was determined by using the Chi-square test for trends.

**Results**: In 1997, there were 149 661 hospital admissions with a principal discharge diagnosis of acute cholecystitis, which increased to 215 995 in 2012 ( *P < *0.001). The mean length of stay for acute cholecystitis decreased by 17% between 1997 and 2012 (i.e. from 4.7 days to 3.9 days; (*P < *0.05). During the same time period, however, mean hospital charges have increased by 195.4 % from US$14 608 per patient in 1997 to US$43 152 per patient in 2012 ( *P < *0.001).

**Conclusion**: The number of inpatient discharges related to acute cholecystitis has increased significantly in the United States over the last 16 years, along with a great increase in the associated hospital charges. However, there has been a gradual decline in the mean length of stay. Inpatient costs associated with acute cholecystitis contribute significantly to the total healthcare bill. Further research on cost-effective evaluation and management of acute cholecystitis is required.

## Introduction

The term “cholecystitis” refers to inflammation of the gallbladder. It may develop acutely in association with gallstones (acute calculous cholecystitis) or, less often, without gallstones (acalculous cholecystitis). More than 80% of people with gallstones are asymptomatic. Acute cholecystitis occurs predominantly as a complication of gallstone disease and typically develops in patients with a history of symptomatic gallstones. Acute cholecystitis develops in 1–3% of patients with symptomatic gallstones [1].

Acute cholecystitis is a syndrome characterized by right upper quadrant pain, fever and leukocytosis associated with gallbladder inflammation. The diagnosis of acute cholecystitis is made on the basis of clinical features such as right upper quadrant pain, fever and leukocytosis and is supported by findings from relevant imaging studies [2]. Treatment is predominantly surgical along with antibiotics, although the timing of surgery is still under debate [3,4]. A meta-analyses of randomized clinical trials in the literature demonstrated that early laparoscopic cholecystectomy (24–72 hours of onset) provides benefit over delayed laparoscopic cholecystectomy (6–12 weeks later) in terms of total hospital stay but conflicting results on conversion rates and postoperative complications [4].

Several studies have shown that gallstone disease constitutes a significant health problem in developed countries [5,6]. Gallstones are prevalent in, 10–15% of the adult population (i.e. 20–25 million Americans have, or will have, gallstones) [7]. The resultant direct and indirect cost of gallbladder disease represents a consumption of about US$6.5 billion annually in the US, constituting a major health burden that has increased > 20% over the last three decades [7]. With an estimated 2.2 million ambulatory care visits each year, gallstone disease is also a leading cause for hospital admissions related to gastrointestinal problems [8]. There is a lack of data about the burden of acute cholecystitis on the US healthcare system; hence, the aim of our study was to analyze the trends in hospitalizations due to acute cholecystitis. 

## Methods

To obtain a population-based estimate of national trends, we used the National Inpatient Sample (NIS) database. The NIS is part of the Healthcare Cost and Utilization Project (HCUP) sponsored by the Agency for Healthcare Research and Quality (Rockville, Maryland). The NIS is the largest publicly available all-payer inpatient care database in the United States and is designed to approximate a 20% sample of US nonfederal hospitals and stratified according to geographic region, ownership, location, teaching status and bed size. The NIS contains data from approximately 8 million hospital stays each year. The 1997 NIS was drawn from 22 states and contains information on all inpatient stays from > 1000 hospitals totaling about 7.1 million records. The 2012 NIS contains discharge data from > 4000 hospitals in 45 states and totals about 8 million records. This large database is an excellent representative sample of the general US population, representing > 95% of the US population, and is useful for analyzing healthcare utilization, access, charges, quality and outcomes [9,10]. The NIS database provides only administrative data for analysis. Patient-specific clinical data are not available.

### Inclusion criteria

To identify cases of acute cholecystitis, we queried the NIS database to recover hospital data on all discharge diagnoses with a primary ICD-9-CM diagnosis code of 574.00, 574.01, 574.30, 574.31, 574.60, 574.6 or 575.0 (acute cholecystitis). The query parameters were configured for the period 1997–2012. NIS data are available from 1988 to 2012; however, there was a change in the NIS dataset in 1997 to include details of patient and hospital characteristics, thereby allowing in-depth analysis of trends over time. Therefore, we chose this particular period for our study. The NIS query provides a graphical user link that enables users to get data on multiple demographic and hospital variables.

### Variables recorded

Patient demographics recorded included age and sex. Hospital characteristics recorded were location (Northeast, Midwest, South and West and metropolitan *vs* non-metropolitan area), type (teaching *vs* non-teaching) and size (small, medium and large). Per HCUPnet definitions, metropolitan areas are those with a population of at least 50 000 people. Areas with a population < 50 000 are the non-metropolitan areas. A hospital is considered to be a teaching hospital if the American Hospital Association Annual Survey indicates it has an American Medical Association (AMA)-approved residency program, is a member of the Council of Teaching Hospitals or has a ratio of full-time equivalent interns and residents to beds of 0.25 or higher. The definition of bed-size varied according to the hospital location and teaching status. The range for small hospitals was 1 to 299 beds. The bed-size range for medium hospitals was 50 to 499, and range for large hospitals was 100 to 500 and more. We also looked at the payer status for all admissions. “Hospital charges” is defined as the amount the hospital charged for the entire hospital stay. It does not include professional (physician) fees. “Aggregate charges” or the "national bill" is defined as the sum of all charges for all hospital stays in the US. “Length of stay” is defined as the number of nights the patient remained in the hospital for this stay.

### Statistical methods

The trends for the annual point estimates of frequency of acute cholecystitis for the data sample were plotted and analyzed. The annual frequency of discharges with acute cholecystitis was computed by dividing the annual number of discharges with acute cholecystitis listed in the NIS database in a given year by the total number of all discharges listed in the NIS for the same year. The temporal trend in frequencies of discharges, lengths of stay, hospital charges and frequencies of deaths in patients with acute cholecystitis was assessed by linear and polynomial regression. The most appropriate functional form for the trend was assessed by examination of regression diagnostic plots. Linear shape was determined for hospital charges and in-hospital deaths: a quadratic shape for length of stay and a cubic shape for number of discharges and discharge rate. *P* *value* < 0.05 was considered statistically significant. All analyses were performed using SAS (version 9.4, The SAS Institute, Cary, NC).

In addition to the percentages available adjacent to the data in the tables, the frequency per 10 000 admissions was also calculated for each categorical variable. These numbers represent the density of patients diagnosed with acute cholecystitis compared with the total number of hospital discharges per category. Each frequency was calculated by dividing the number of patients with acute cholecystitis by the total discharges in a specific categorical variable for each year and multiplying that number by 10 000. We viewed the counts as arising from a Poisson distribution and the total discharges as an offset, yielding Poisson rates that were compared over time using Poisson regression and yielded relative rates (RRs) and 95% confidence intervals (CIs) that expressed the ratio of rate per 10 000 in 2012 to that of 1997. These values differed from the percentages, which describe each category exclusively for either patients with acute cholecystitis or for total discharges. The percentages distinguished differences among the variables for each specific year, whereas the frequencies were vital for comparing trends from 1997 to 2012, especially for age group and region.

## Results

### Number and cost of acute cholecystitis discharges

**Figure 1.**
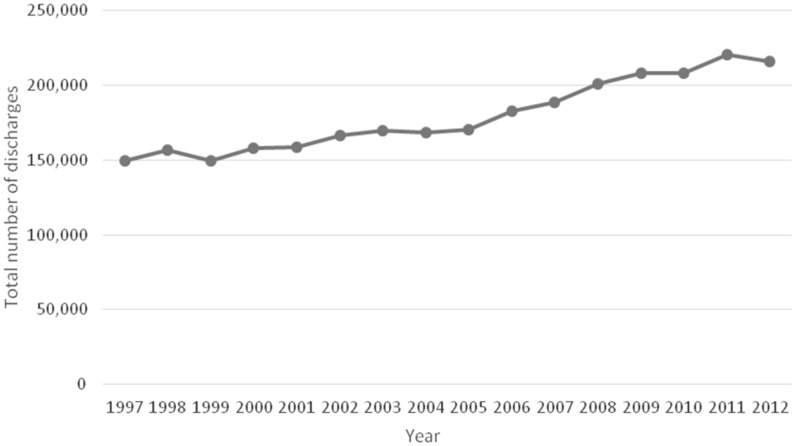
The number of hospital discharges with a principal diagnosis of acute cholecystitis during the period from 1997 to 2012.

From 1997 to 2012, the total number of hospital discharges with a principal diagnosis of acute cholecystitis increased by 44.3% from 149 661 to 215 995 (*P* < 0.001) (Figure 1). The number of hospital discharges with acute cholecystitis as either a principal or a secondary diagnosis increased from 183 570 to 272 130 during the same time period.

**Figure 2.**
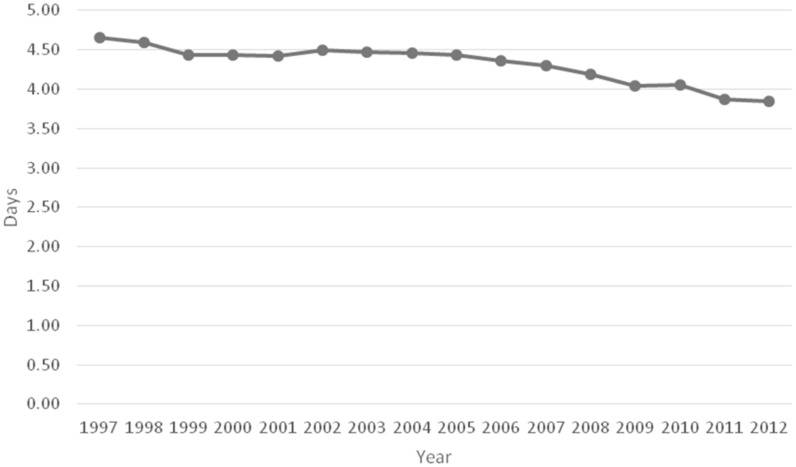
The mean length of hospital stay of patients with a principal diagnosis of acute cholecystitis during the period from 1997 to 2012.

 The frequency of hospital discharges for acute cholecystitis as a principal diagnosis increased from 45 per10 000 discharges to 76.1 per 10 000 discharges. This increase reached statistical significance (RR = 1.68, 95%CI: 1.67–1.7; *P* < 0.001). The average length of hospital stay for patients with acute cholecystitis decreased slightly from 4.7 days to 3.9 days ( *P < *0.05) between 1997 and 2012 (Figure 2).

The aggregate costs of hospital visits in which acute cholecystitis was the primary discharge diagnosis increased 327 % from US$2 184 774 455 in 1997 to US$9 318 972 919 in 2012. The portion of the national bill (total aggregate charges for acute cholecystitis/ total national bill) for acute cholecystitis discharges increased from 0.58% in 1997 to 1.52 % in 2012.

**Figure 3.**
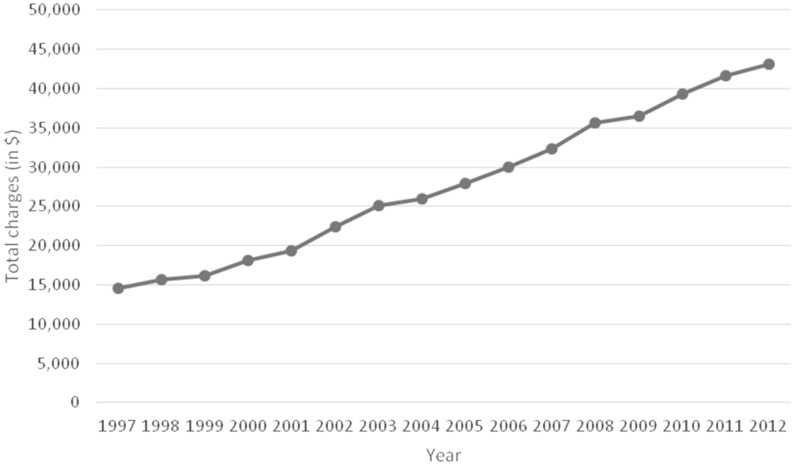
The average total hospital charges per hospitalization for primary diagnosis of acute cholecystitis during the period from 1997 to 2012.

Despite the decrease in the average length of stay, the mean total charges for acute cholecystitis-related hospital admissions increased substantially between 1997 and 2012. Mean hospital charges per patient increased 195.4% from US$14 608 in 1997 to US$43 152 in 2012 with a statistically significant linear trend (*P* < 0.001) indicating a simple linear increase in charges over this period (Figure 3).

### Patient characteristics by age

The highest rate of discharges in 1997 was in the age group of 65–84 years, while the highest rate of discharges in 2012 was in the age group of 18–44 years. The increase in frequency of discharge rates was most remarkable in the age group of 18–44 years, in which it increased almost three times from 1997 to 2012 (RR = 2.94, 95%CI: 2.92–2.97; *P* < 0.001), followed by the 1–17 years age group, in which the frequency more than doubled (RR = 2.18, 95%CI: 2.04–2.34; *P* <0.001]. Although the increased in acute cholecystitis discharge rates in the 45–64 years (RR = 1.08, 95%CI: 1.07–1.09), 65–84 years (RR = 1.15, 95%CI: 1.14–1.16) and 85+ years (RR = 1.2, 95%CI: 1.17–1.23) age groups were lower, they also reached statistical significance.

### Patient characteristics by sex

The frequency of acute cholecystitis was greatest in women in both 1997 and 2012, increasing from 47.86 per 10 000 discharges in 1997 to 86.11 per 10 000 discharges in 2012 (RR = 1.79, 95%CI: 1.78–1.81; *P < *0.001). The increase for men went from 40.99 per 10 000 discharges in 1997 to 64.88 per 10 000 discharges in 2012 (RR = 1.58, 95%CI 1.56–1.6; *P < *0.001).

### Patient characteristics by payer group

The relative frequency of acute cholecystitis discharges increased for all types of payer groups over the 16-year span. The highest absolute number of acute cholecystitis discharges was in the private insurance group in both 1997 and 2012 (RR = 1.99, 95%CI: 1.97–2.01; *P* < 0.001). The relative frequency of patients with acute cholecystitis using Medicaid as a form of payment increased by 221% from 24.6 per 10 000 discharges in 1997 to 79 per 10 000 discharges in 2012 (RR = 3.19, 95%CI: 3.13–3.26; *P < *0.001). This increase was greater than three folds, which was the highest among all forms of payment, followed by private insurance (50.9 per10 000 in 1997 to 102 per10 000 in 2012) and uninsured groups (62.7 per10 000 in 1997 to 152.7 per10 000 in 2012),which more than doubled.

### Acute cholecystitis discharges by hospital characteristics and region

Metropolitan areas had higher absolute numbers of acute cholecystitis discharges than non-metropolitan areas in both 1997 and 2012. However, non-metropolitan areas had higher relative frequencies of acute cholecystitis discharges in 1997 and 2012. The frequency of discharges for metropolitan areas increased from 41.91 per 10 000 in 1997 to 75.26 per10 000 in 2012 (RR = 1.79, 95%CI: 1.78–1.80; *P < *0.001). For non-metropolitan areas, the frequency of discharges increased from 62.1 per 10 000 in 1997 to 82.3 per 10 000 in 2012 (RR = 1.32, 95%CI: 1.30–1.34; *P < *0.001).

The South had the highest absolute number of both acute cholecystitis discharges and total discharges in 1997 as well as in 2012. The South also had the highest frequency of discharges in 1997 with 47.5 per 10 000 discharges followed by the Northeast with 45.3 per10 000 discharges and the West with 44.6 per10 000 discharges. In 2012, the West had the highest frequency of discharges with 101.6 per10 000 followed by Northeast with 75.8 per10 000 and the South with 73 per10 000 discharges. The frequency of acute cholecystitis discharges more than doubled in the West (RR = 2.27, 95%CI: 2.24–2.30; *P < *0.001), whereas the increase in all other regions was smaller but still statistically significant ( *P < *0.001).

In 1997, patients with acute cholecystitis were more likely to be diagnosed in a hospital with a small number of beds (51.9 per10 000 discharges, *P < *0.001), whereas patients with acute cholecystitis were more likely to be diagnosed from a hospital with a medium number of beds (81.1 per 10 000 discharges, *P < *0.001) in 2012.

## Discussion

Our study found that acute cholecystitis is a growing problem in the United States and an increasing burden on the healthcare system. We found that there was a significant increase in acute cholecystitis-related discharges and associated hospital costs over our study period. The cost related to hospitalizations with acute cholecystitis as the primary diagnosis in 2012 was over US$9.3 billion.

Between 1997 and 2012, the frequency of acute cholecystitis-related discharges as a primary diagnosis increased by 44.3%. This increase was likely due to multiple factors such as increasing use of anti-cholesterol medications and the rise in the incidence of diabetes and obesity. These factors lead to an increased susceptibility for gallstones and thereby increase the risk of developing acute cholecystitis. However, it is interesting to note that the average length of stay decreased from 4.7 days to 3.9 days during the same period, which was likely due to laparoscopic cholecystectomy replacing open cholecystectomy as the standard treatment modality for acute cholecystitis. In 2006, there were approximately 503 000 laparoscopic cholecystectomies performed [11]. Laparoscopic cholecystectomy is superior to open cholecystectomy as a treatment for acute cholecystitis because of a lower incidence of complications, shorter length of postoperative hospital stay, faster recovery and earlier return to work [3,12].

The average total charges per patient increased by 195.4%; likewise, the overall percentage of total hospital costs (the national bill) associated with acute cholecystitis more than doubled during this time period from 0.58 to 1.52%.

As laparoscopic cholecystectomy has gained wider acceptance, complications that were rarely seen with open cholecystectomy (e.g. bile duct injuries) are more commonly reported in as many as 5% of patients. At present, approximately 750 000 laparoscopic cholecystectomies are performed annually in the United States (accounting for roughly 90%t of all cholecystectomies) with an overall serious complication rate that remains higher than that of open cholecystectomy despite increasing experience with the procedure [13,14] (possibly adding further to the overall hospitalization costs and length of stay).

We also analyzed the data on the basis of patient characteristics of age, sex and payer status. The frequency of hospital discharges with regard to age increased for all age groups from 1997 to 2012. The trend for the highest rate of discharges for age group shifted from 65–84 years in 1997 to 18–44 years in 2012. Obesity has been well-recognized for its association with gallbladder disease [15,16]. The number of obesity-related admissions also increased from approximately 820 000 in 1997 to 3.7 million in 2012. This shifting trend towards the 18–44 age group may be due to increasing rates of obesity and decreased physical exercise among the younger populations in the United States [17,18].

According to the Framingham study, which examined the risk factors for cholelithiasis in a 10-year follow-up study of 30–59-year-old subjects, the risk of cholelithiasis within 10 years was highest among the 55–62-year-old age group, and most of the patients were diagnosed with cholelithiasis in their fifties and sixties [19,20]. Although the incidence of cholelithiasis in female patients of all age groups is greater than twice that of male patients, the Framingham study also found that the sex difference tends to shrink with increasing age [19].

In our study, the rate of hospitalizations was significantly higher in women as compared with men. Women constituted almost 60% of all hospital discharges with the diagnosis of acute cholecystitis in 1997 as well as in 2012. In our study, 90% of the acute cholecystitis admissions were due to cholelithiasis/choledocholithiasis in 1997, while it was approximately 81% in 2012, suggesting an increase in acalculous cholecystitis as well. The Framingham study also confirmed that cholelithiasis patients tend to be more obese than non-cholelithiasis patients [19]; however, there is a report that this tendency is much more prominent in female than in male patients [20].

In terms of payer status, we found that the highest absolute number of acute cholecystitis discharges was in the private insurance group in both 1997 and 2012. Also notable was the fact that the relative frequency of patients with acute cholecystitis using Medicaid as a form of payment increased more than three times, which was the highest of all forms of payment. This significant increase may be secondary to these patients being less likely to seek care at the onset of a health concern because of limited or hindered access to healthcare [21]. This would result in waiting until a health concern becomes severe or debilitating before seeking treatment and thus a higher likelihood of being admitted as in-patients. Other reasons could be the increasing premiums of the private health insurance companies, which are becoming less affordable to lower income people. Also, the economic crisis resulting in people losing their jobs could be another explanation for this finding. As outpatients, surgical patients with private insurance are often able to obtain personal referral to more experienced surgeons, while patients with Medicaid are most often referred to a clinic system with less specialized surgeons or self-refer to the emergency department [22]. Studies have shown that Medicaid populations tend to receive the majority of their primary care within the emergency departments [23]. Patients with Medicaid who present to the emergency department with acute cholecystitis are less likely to receive cholecystectomy for their condition during that initial hospital visit and have slightly worse surgical outcomes compared with those having private insurance. This disparity is most likely due to a combination of factors including the healthcare system and cultural factors [22].

We also analyzed the data with respect to hospital characteristics such as metropolitan/non-metropolitan areas, region and hospital-bed size. We found that metropolitan areas had higher absolute numbers of acute cholecystitis discharges than non-metropolitan areas both in 1997 and 2012. This could be due to the fact that metropolitan areas are more densely populated than non-metropolitan areas. We also found that patients with acute cholecystitis in 1997 were more likely to be diagnosed in a hospital with a small bed-size, whereas patients with acute cholecystitis in 2012 were more likely to be diagnosed in a hospital with a medium bed-size.

In terms of regional distribution, the South had the highest number of absolute discharges of acute cholecystitis patients in both 1997 and 2012 as well as the highest frequency of discharges. In 2012, the West had the highest frequency of discharges, which have more than doubled since 1997. The findings of our study were similar to the study by Zafar *et al.*, which also stated that the highest number of discharges of patients with acute cholecystitis was in the South [24]. In that study, large numbers of patients were in metropolitan areas, and the majority of them were admitted to hospitals with large bed-size, similar to the findings in our study.

The design of this study and the nature of the NIS data set include some important limitations. As this is an administrative data set, it is reflective of the coding practices of each healthcare institution. It is likely that these results underestimate the actual incidence of acute cholecystitis discharges because patients’ discharges may have been coded with an alternative diagnosis such as abdominal pain. In addition, this data set does not control for errors during data entry. Also, individual patient-specific clinical information (e.g. race or the procedures performed on the patient) was not obtainable and thereby limited comments to the given demographics of the research sample. Future studies analyzing patient-specific trends and individual hospital coding practices may provide additional clarification of the information in this study. Importantly, the NIS data set does not provide sufficient patient and hospital details to determine the factors that could potentially explain the significant rise in hospital discharges and their costs.

In conclusion, acute cholecystitis is an escalating concern to the United States healthcare system. This is demonstrated by the significant increase in the frequency of acute cholecystitis-related discharges and their associated hospital costs between 1997 and 2012. Future studies analyzing the treatment and diagnostic practices of physicians treating acute cholecystitis and studies that investigate preventative measures are necessary steps for reducing the burden of acute cholecystitis.

*Conflict of interest statement*: none declared.

**Table 1.** Number and frequency of discharges with acute cholecystitis (AC) by patient and hospital characteristics in 1997 and 2012

Category	Categorical variable	AC in 1997, N (%)	AC in 2012, N (%)	Total admissions in 1997, N (%)	Total admissions in 2012, N (%)	AC per 10 000 admissions in 1997	AC per 10 000 admissions in 2012
All discharges		149 661 (100)	215 995 (100)	33 230 554 (100)	28 391 049 (100)	45.04	76.08
Age group (y)	1–17	1251 (1)	2130 (1)	1 766 699 (5)	1 376 026 (5)	7.08	15.48
	18–44	45 229 (30)	73 150 (34)	9 074 102 (27)	4943137 (17)	49.84	147.98
	45–64	45 117 (30)	69 995 (32)	6 266 540 (19)	9 003 812 (32)	72	77.74
	65–84	50 254 (34)	58 265 (27)	9 644 446 (29)	9 724 120 (34)	52.11	59.92
	85+	7806 (5)	12 430 (6)	2 245 807(7)	2 980 584 (11)	34.76	41.7
Sex	Male	55 878 (37)	87 065 (40)	13 632 763 (41)	13 4189 72 (47)	40.99	64.88
	Female	93 771(63)	128 910 (60)	19 593 669 (59)	14 969 687 (53)	47.86	86.11
Payer	Medicare	55 347 (37)	73 880 (34)	12 070 265 (36)	14 227 410 (50)	45.85	51.93
	Medicaid	13 418 (9)	31 040 (14)	5 448 491 (16)	3 926 823 (14)	24.63	79.05
	Private insurance	65 246 (44)	75 080 (35)	12 814 864 (39)	7 360 684 (26)	50.91	102
	Uninsured	10 130 (7)	27 180 (13)	1 615 542 (5)	1 779 681 (6)	62.7	152.72
	Other	4974 (3)	8305 (4)	1 201 195 (4)	1 026 506 (4)	41.41	80.91
Median income for zip code	Low	–	61 170 (28)	–	8 646 773 (30)	–	70.74
	Not low	–	150 360 (70)	–	19 070 345 (67)	–	78.84
Hospital owner	Government	20 840 (14)	25 675 (12)	4 707 979 (14)	3 390 284 (12)	44.27	75.73
	Private, not-for-profit	104 754 (70)	154 070 (71)	23 924 271 (72)	20 916 148 (74)	43.79	73.66
	Private, for-profit	23 568 (16)	36 250 (17)	4 464 111 (13)	4 084 618 (14)	52.79	88.75
Location	Non-metropolitan	32 079 (21)	26 950 (12)	5 165 442 (16)	3 273 738 (12)	62.1	82.32
	Metropolitan	117 083 (78)	189 045 (88)	27 930 919 (84)	25 117 311 (88)	41.92	75.26
Bed-size	Small	27 413 (18)	32 350 (15)	5 284 288 (16)	4 118 586 (15)	51.88	78.55
	Medium	51 986 (35)	60 165 (28)	11 047 425 (33)	7 416 819 (26)	47.06	81.12
	Large	69 764 (47)	123 480 (57)	16 764 648 (51)	16 855 643 (59)	41.61	73.26
Region	Northeast	30 720 (21)	42 855 (20)	6 784 405 (20)	5 652 002 (20)	45.28	75.82
	Midwest	31 899 (21)	39 860 (18.5)	7 740 346 (23)	6 525 448 (23)	41.21	61.08
	South	58 828 (39)	80 500 (37)	12 373 424 (37)	11 019 844 (39)	47.54	73.05
	West	28 214 (19)	52 780 (24.5)	6 332 379 (19)	5 193 755 (18)	44.56	101.62
